# Attention Deficit Associated with Early Life Interictal Spikes in a Rat Model Is Improved with ACTH

**DOI:** 10.1371/journal.pone.0089812

**Published:** 2014-02-24

**Authors:** Amanda E. Hernan, Abigail Alexander, Pierre-Pascal Lenck-Santini, Rod C. Scott, Gregory L. Holmes

**Affiliations:** 1 Department of Neurology, Geisel School of Medicine at Dartmouth, Hanover, New Hampshire, United States of America; 2 Institute of Child Health, University College London, London, United Kingdom; 3 Department of Neurological Sciences, University of Vermont College of Medicine, Burlington, Vermont, United States of America; University of Modena and Reggio Emilia, Italy

## Abstract

Children with epilepsy often present with pervasive cognitive and behavioral comorbidities including working memory impairments, attention deficit hyperactivity disorder (ADHD) and autism spectrum disorder. These non-seizure characteristics are severely detrimental to overall quality of life. Some of these children, particularly those with epilepsies classified as Landau-Kleffner Syndrome or continuous spike and wave during sleep, have infrequent seizure activity but frequent focal epileptiform activity. This frequent epileptiform activity is thought to be detrimental to cognitive development; however, it is also possible that these IIS events initiate pathophysiological pathways in the developing brain that may be independently associated with cognitive deficits. These hypotheses are difficult to address due to the previous lack of an appropriate animal model. To this end, we have recently developed a rat model to test the role of frequent focal epileptiform activity in the prefrontal cortex. Using microinjections of a GABA_A_ antagonist (bicuculline methiodine) delivered multiple times per day from postnatal day (p) 21 to p25, we showed that rat pups experiencing frequent, focal, recurrent epileptiform activity in the form of interictal spikes during neurodevelopment have significant long-term deficits in attention and sociability that persist into adulthood. To determine if treatment with ACTH, a drug widely used to treat early-life seizures, altered outcome we administered ACTH once per day subcutaneously during the time of the induced interictal spike activity. We show a modest amelioration of the attention deficit seen in animals with a history of early life interictal spikes with ACTH, in the absence of alteration of interictal spike activity. These results suggest that pharmacological intervention that is not targeted to the interictal spike activity is worthy of future study as it may be beneficial for preventing or ameliorating adverse cognitive outcomes.

## Introduction

It is widely believed that frequent epileptiform events seen in children with epilepsy are capable of causing deleterious alterations in developing brain networks and are therefore associated with the high incidence of cognitive deficits and psychiatric comorbidities in these patients [1], [2]. It has been suggested that when the EEG is normalized, these impairments are often reduced or eliminated [3], [4]. This line of thinking has led to the idea that, like overt seizures, EEG interictal discharges should also be treated [5]. However, it is unknown if the epileptiform activity itself, some downstream consequence of the epileptiform activity, or a combination of both of these mechanisms negatively impacts cognition in the developing brain.

We have recently shown selective deficits in measures of attention and sociability in adult animals with a history of interictal spikes (IIS) in the prefrontal cortex (PFC) during development. The PFC was chosen based on the preponderance of PFC-related comorbidities in children with pediatric epilepsy. We hypothesized that pharmacological treatment could improve the IIS activity and that this treatment could also ameliorate these associated behavioral deficits.

The drug H.P. Acthar gel was chosen to test these hypotheses. The primary active compound in Acthar gel is adrenocorticotrophic hormone (ACTH), which is part of the *hypothalamic-pituitary-adrenal* (HPA) axis and leads to downstream release of cortisol in humans. ACTH is commonly used in epilepsies with frequent IIS and has been shown to decrease seizure frequency and normalize the EEG [6]–[9]. Treatment with ACTH in our model could therefore lead to increased attentiveness during a behavioral task and improvements in social behavior. Interestingly, we show that ACTH administration modestly, but significantly, ameliorates the attention deficit in animals with a history of IIS, but it does so without alteration of IIS activity itself. The mechanism for this is yet unknown, and ACTH does not seem to be effective in improving social behaviors in our model. Understanding when and how ACTH is effective in improving cognition may lead to new treatments for cognitive deficits associated with pediatric epilepsy that are independent of the treatment of seizures.

## Materials and Methods

### Ethics Statement

All experiments were performed in strict accordance with the guidelines set down by the National Institutes of Health and the Geisel School of Medicine at Dartmouth for the humane treatment of animals. The animal protocol was approved by the Institutional Animal Care and Use Committee of Dartmouth College (IACUC protocol number 09-08-01). All surgeries were performed under isoflurane anesthesia (2–3% in oxygen) and 5 mg/kg ketoprofen was used as an analgesic post-operatively. All efforts were made to minimize animal pain and utilize the smallest number of animals necessary to achieve the goal of the study.

### Animals

Animals were housed 2 animals per cage on a 12∶12 light: dark cycle with access to standard rat chow and water *ad libitum* unless otherwise noted. Behavioral data presented herein are pooled from three experimental cohorts of animals. The first cohort, containing only a control (N = 5) and an untreated IIS group (N = 5) and an additional two cohorts contain a control group (N = 4), an untreated IIS group (N = 4) and a treated IIS group (N = 8).

### Overview of Study Design

**Figure 1:**
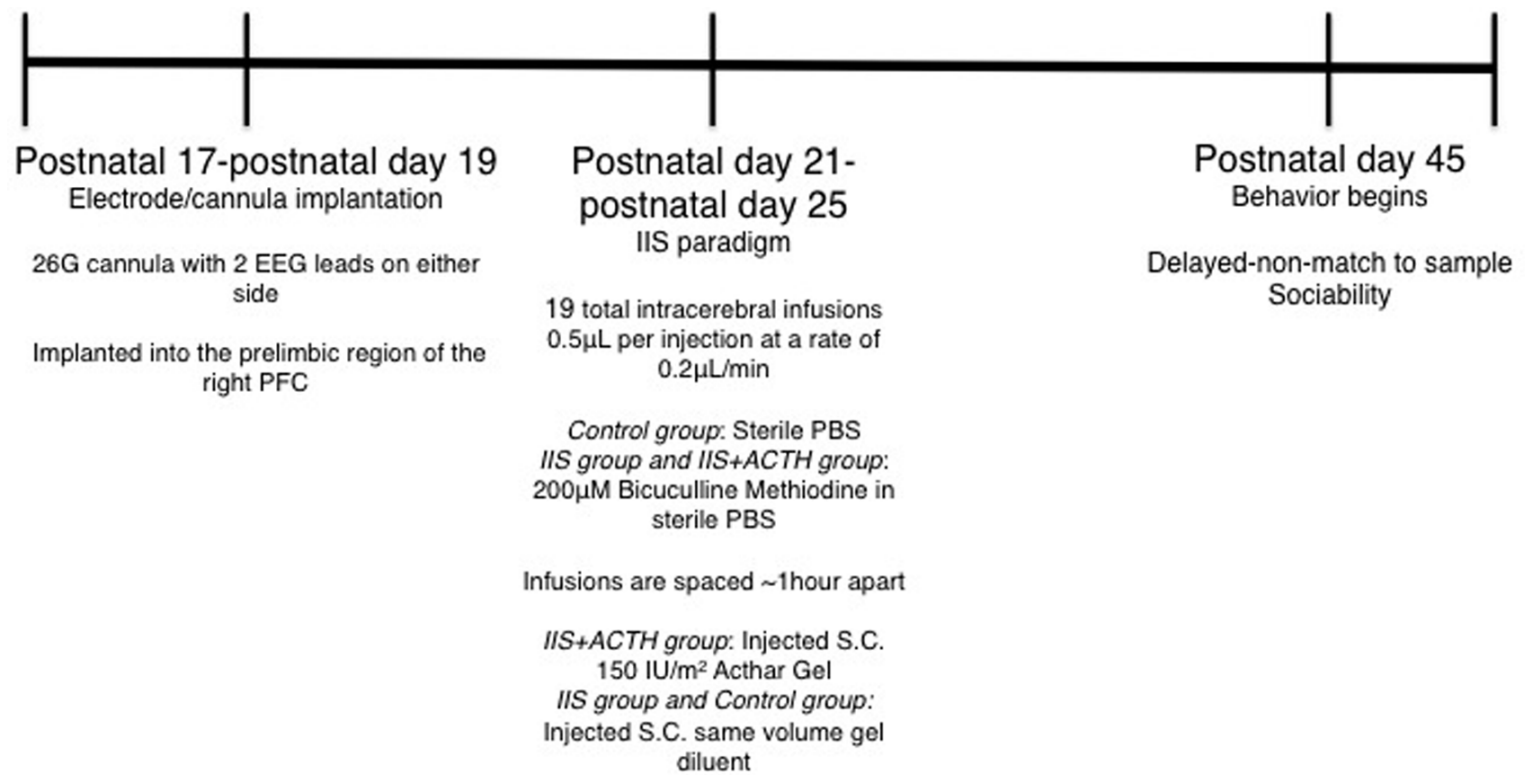
Experimental design. Animals were implanted at p17, p18 or p19 and allowed to recover for 2–5 days before the early life IIS began. Early life IIS took place from p21–p25 with infusions of bicuculline methiodine or saline, 3–4 infusions per day spaced 1 hour apart. Three groups of animals were used, a control infused with sterile PBS and two IIS groups infused with BMI, one treated with ACTH and one untreated. Control and untreated IIS groups received subcutaneous vehicle injections at the same time as ACTH group received ACTH. EEG was recorded immediately post BMI injection in both IIS groups. All behavioral experiments were conducted from p45 onward in order to evaluate the long-term effect of ACTH on IIS-associated behavioral deficits.

See Figure 1 for full experimental design. Animals were chronically implanted unilaterally in the right PFC with a cannula attached to two EEG leads. Saline or a GABA_A_ antagonist (bicuculline methiodine) was infused through the cannula multiple times per day for five consecutive days during development and local EEG was recorded at the time of infusion. Bicuculline-injected animals developed interictal spike (IIS)-like events on the EEG, with minimal seizure involvement. The long-term effect of treatment with ACTH on later PFC-related cognition was examined.

### Surgery

Sprague-Dawley male rat pups (Charles River Laboratories, Wilmington, MA) age p17, p18 or p19 (Fig. 1) were implanted with custom-made cannula/EEG electrodes (Plastics One, Roanoke, VA). Each implant had a 26 G guide cannula fitted with a 33 G dummy cannula. The guide and injection cannula measured 6 mm in length, the dummy cannula was 6.2 mm in length. Affixed to either side in the medial/lateral direction were two EEG wires, 7 mm each in length, which were attached to a Mill-Max connector (Mill-Max Manufacturing Corporation, Oyster Bay, NY). The electrode/cannula set up was implanted into the right prefrontal cortex so that the tip of the guide cannula sat at the following coordinates; 3.0 mm anterior to bregma, 0.8 mm to the right of midline, 2.5 mm ventral to the surface of the brain. Implants were held in place with grip cement (DentSply International, York, PA). All animals were treated with 5 mg/kg ketoprofen every 4 hrs as needed during the post-surgical recovery phase.

### Early-life Epileptiform Activity Model

After a 2–4 day recovery period, animals were injected intracortically with 0.5–0.7 µL of 200 µM bicuculline methiodine (BMI) using a pump that delivered fluid unilaterally to the right PFC at 0.2 µL/minute. From p21–p25, animals were injected a total of 19 times. Three intracerebral infusions were made on the first day, with four infusions made per day on each of the four days thereafter. Injections were separated by approximately 1 hour. Littermate controls were also chronically implanted with an electrode/cannula and injected with sterile saline at the same time as BMI in the IIS group (Fig.1). Continuous intracranial EEG was recorded after each BMI infusion from a location adjacent to the injection site and referenced to a common cerebellar electrode. Recordings were made for 25–30 mins in order to ensure IIS activity was achieved. Presence of behavioral and electrographic seizures was visually monitored in real time and video recorded. Spike width and spike rate were calculated semi-automatically using a MATLAB (Mathworks, Torrance, CA) function that identified putative spike events in an EEG trace. EEG containing putative spike events were separated from the entire trace and visually confirmed as true events or artifact on the basis of shape and kinetics. Width of true spike events was calculated using a MATLAB function that measured time from peak to trough.

### ACTH

IIS and littermate control animals were injected subcutaneously with either vehicle or H.P. Acthar Gel (ACTH, obtained from Questcor pharmaceuticals). Vehicle control gel was also obtained from Questcor pharmaceuticals and had the same composition as Acthar diluent. ACTH injections were made once per day, 1 hr before BMI injections began. A dose of 150 IU/m^2^ (international units per meter squared) was injected. Subcutaneous injection was chosen to maximize duration of release of the drug. Body surface area (BSA) was estimated as follows: surface area (in cm^2^) = 9.1× (mass in grams)^2/3^ [10]. BSA measurements per cm^2^ were multiplied by a factor of.0001 to obtain BSA in m^2^. BSA measurements (in m^2^) were then multiplied by the 150 IU/m^2^ to obtain a daily dose in IU. Acthar Gel was provided in 5 mL vials containing 80 IU of ACTH per mL. N = 8 animals were treated with ACTH, N = 8 animals were treated with vehicle.

### Delayed Non-match to Sample (DNMS)

An operant box (Med Associates Inc., St. Albans, VT) enclosed in a dimly lit, sound-attenuating chamber was used for delayed non-match to sample (DNMS) experiments. Inside the box, two retractable levers were located on one wall separated by a pellet dispenser. The opposite wall contained a nose-poke hole. Stimulus lights were located above each lever, the food cup and the nose-poke hole. Food pellets were used as rewards (45 mg purified rodent dustless precision food pellet F0021; Bio-Serv, Frenchtown, NJ). Animals were food deprived to approximately 85–90% of predicted weight according to the growth chart provided by Charles River Laboratories for Sprague-Dawley rats. During this time, each animal received 14.5 g of food per day, in addition to any food pellets rewarded during the task. DNMS sessions were done one session per day, 7 days a week at approximately the same time each day.

The DNMS task involved several training steps before a full trial could be executed. Rats were initially trained to associate both freely available levers with food reward (labeled “PR1”). They were then trained to press only one extended lever (“Lever Press”), and subsequently trained to place their snout in the nose hole when the light above it was illuminated. This last training part was first performed while the operant box lights were switched off entirely to encourage the rat to follow the illuminated light about the nosepoke hole (“Nosepoke Dark”) and then in a dimly-lit operant box (“Nosepoke Light”). Criteria for advancing through these training sessions were as follows; bar presses: >25 rewards received, with both levers pressed at least once each; nose-poke dark: >25 rewards received, nose poke light: >10 rewards. If <10 rewards were given in a session, rats were demoted to the previous session stage.

A final training session was a forced lever-nosepoke-opposite lever session (“L-NP-L”). The rat was presented with one lever (sample phase), had to press the lever, break the nose-poke beam in the opposite wall, and was then presented only the lever (test phase) that was not presented before (non-match lever). A successful press of the test lever in less than 10 s was associated with a food reward, or else the trial would reset.

Once animals were able to perform this type of forced trial with an 80% response rate (>40 trials), they advanced to “DNMS training” trials, which contained the full DNMS paradigm with a 0 s delay. Here rats were presented with both test levers and had to press the one that was not presented in the sample phase. During this training paradigm, an error during one trial (i.e., the rat chose the match lever instead of the non-match lever) resulted in a subsequent forced L-NP-L as in the previous training step. Once criterion (80% correct on the DNMS trials) was achieved within the DNMS training session, full DNMS sessions began.

DNMS was done in blocks with increasing delay times during the nosepoke phase; block 0 had a 0 s delay, block 1 had trials with randomly assigned 0–5 s delays, and block 2 had trials with randomly assigned 0–10 s delays. Criteria to pass to the next block include accuracy of 80% or more (40 or more correct trials out of a total of 50 trials per session) during the session and no clear lever preference. Lever preference or less than 60% accuracy (fewer than 30 correct trials) resulted in regression to the previous block or phase of DNMS training. Behavioral software (Med Associates) gave output measures on correct and incorrect trials, as well as errors of omission: the former relating to the working memory component of the task and the latter relating to attention during the task. An error of omission was counted when a response was expected from the animal and the animal failed to respond within 10 s.

### Sociability

The sociability experiment was based on a previously described mouse sociability task [11]. In brief, animals were placed in a plexiglass box 1.22 m (48″) in length and 0.41 m (16″) in height that was separated into three smaller chambers; a central chamber measuring 0.25 m (10″) in width, with adjacent right and left chambers measuring 0.47 m (18.5″) in width each that were separated from the center chamber by a plexiglass wall with a removable door cutout. After a 5-min acclimation phase in the center chamber, animals were allowed to roam freely between the left and right chamber containing a novel object and a novel age-matched male rat, respectively for a 10-min test session. Both the novel object and novel rat were enclosed in a mesh container with openings large enough for only the nose of the animal in order to ensure social approach was initiated by the test rat and not the novel rat. The primary outcome measure in this task was the time spent in the chamber with the novel rat vs. the time in the novel object chamber.

### Statistics

Statistics were done using Prism (GraphPad, La Jolla, CA) and SPSS v.20 (IBM Armonk, NY). We carried out multivariable repeated measures regression analysis using generalized estimating equations in order to assume the correct distribution for the data. IIS (bicuclline administration) and ACTH administration were both modeled as predictors. The effects of litter and seizures were also tested and were not significant in any of our experiments; the effect of session number was modeled in our DNMS data set and was not significant. The effect of ACTH on IIS-width and average spike rate and all histological data was analyzed using a two-tailed Student’s t-test, the effect of ACTH on IIS rate over time was assessed using a repeated-measures ANOVA.

## Results

### IIS Model

**Figure 2:**
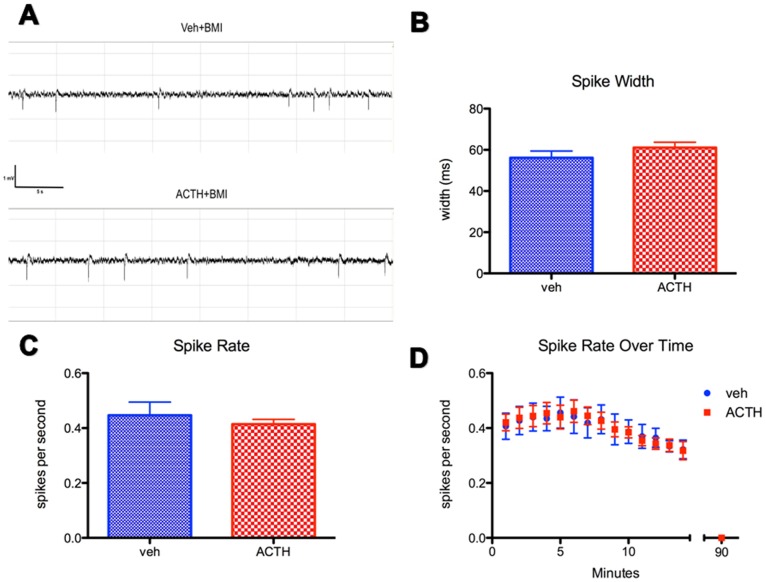
The effect of ACTH IIS activity. ACTH (**A, top trace**) does not have any effect on IIS activity when compared with vehicle-injected (**A, bottom trace**) IIS. Both EEG traces are taken from recordings made on the second day of IIS, 10-mins after the initial BMI injection. ACTH does not have any effect on spike width (**B**) or spike rate (**C**) or spike rate over time (**D**) during the 20-min post-injection EEG recording session. Bars represent mean ± SEM, N = 4 IIS, N = 8 IIS+ACTH.

IIS activity began immediately after BMI microinjection and lasted for 10–20 mins post-injection while the animal was awake (Fig. 2D). Saline-injected control animals never developed IIS. All animals were monitored in real time during the 25–30 min recording period post-injection. IIS activity had a mean rate of 0.45±0.2 spikes/s in vehicle controls, and 0.41±0.08 spikes/s in ACTH-treated animals (Fig. 2C). This rate was independent of injection number (p = 0.41).

Brief seizures lasting less than 15 s (average length 14.9±0.3 s) occurred in 8 of 17 BMI-exposed animals. There was no difference in seizure activity between groups exposed to the ACTH and vehicle-treated IIS animals. Overall frequency was 0.22±0.10 seizures per day. Presence or absence of seizures was modeled into all statistics and there was no significant effect of seizures in any of our behavioral outcomes.

### The Effect of ACTH on BMI-induced IIS

To determine whether or not behavioral deficits seen in animals with a history of IIS could be ameliorated with pharmacological treatment, animals were injected subcutaneously with ACTH one hour before beginning BMI injection. ACTH injection did not alter spike width, the frequency or duration of IIS as measured during the first 20 mins post-injection EEG recording period (Fig. 2B, C, D). Qualitatively, EEG from animals treated with ACTH appears similar to EEG from vehicle-injected controls (Fig. 2A).

### The Effect of ACTH on IIS-induced Behaviors

**Figure 3:**
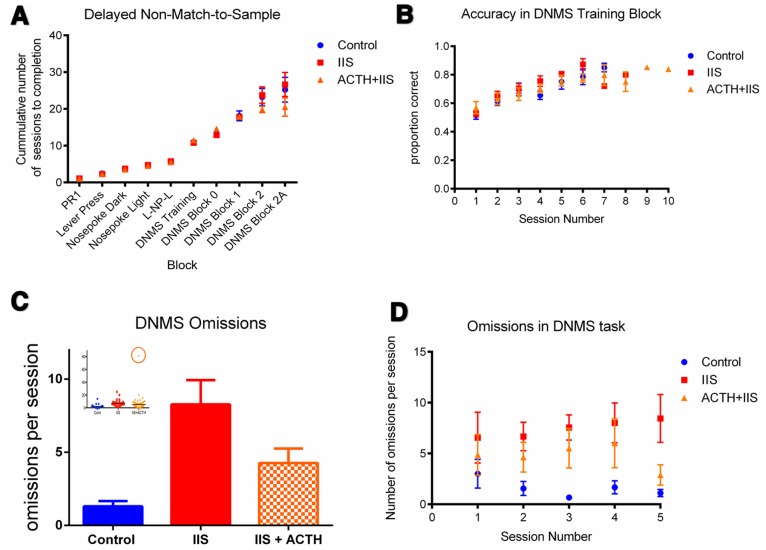
Improvements in attention after ACTH administration. Working memory as measured by cumulative number of sessions to task completion (**A**) and accuracy in the training session block (**B**) in the DNMS task were not different after IIS, or with treatment with ACTH. However, IIS animals (red) made significantly more omissions per session in the task than their control (blue) counterparts, and this was significantly improved with ACTH treatment (orange) (p<0.05) (**C**). Inset in (**C**) shows the presence of an outlier (circled) in the ACTH+IIS (orange) group. The number of omissions errors per day did not vary based on the difficulty in the working memory component of the task (**D**). Data points on all graphs represent mean ± SEM, N = 9 Control, N = 9 IIS, N = 8 IIS+ACTH.

A delayed non-match to sample (DNMS) task was chosen in order to test for the presence of deficits in working memory and attention. During the DNMS task, all animals were fed 14.5 g of food per day; weights did not significantly differ between the treated and untreated IIS groups during the DNMS experiment (group mean daily weight: 238.3±7.1 g in control group, 228.3.9±5.7 g in IIS group, 214.7±2.5 g in IIS+ACTH group; p>0.05). Additionally, number of rewards eaten on the first day of the task, when rewards are freely available with the press of either of both continuously extended levers during a fixed 20-min session, were not different between the groups (28.6±2.6 pellets in controls, 34.6±4.5 pellets in IIS animals, 38.8±5.9 pellets in IIS+ACTH animals; p = 0.29). Taken together, all of these data indicate that rats in all groups were equally motivated to complete the task. We found no significant difference in number of sessions to block completion (Fig. 3A), or in accuracy by day during any of the blocks (accuracy in the DNMS training block show in Fig. 3B), suggesting that working memory is largely intact in IIS animals and did not differ in IIS+ACTH animals.

However, animals with a history of IIS did make significantly more errors of omission than control animals (1.3±0.4 in controls vs. 8.3±1.7 in IIS). Treatment with ACTH decreased the number of omissions errors IIS animals made during the DNMS task. Errors of omission have previously been interpreted as a deficit in attention during the task.

On day one of the DNMS task, one animal in the ACTH+IIS group made 82 total omissions in one session, four times more omissions than were ever observed in any other session from any animal in the cohort. This session is significantly influencing the data and is a statistical outlier (Dixon’s Q test, p<0.002, shown in circle in inset in Fig. 3C). For this reason this single session was eliminated from the analysis. The ACTH+IIS group averaged 4.26±0.99 omissions per session, a significant decrease from the IIS group (p = 0.03, Fig. 3C), however with the outlier session included in the analysis the ACTH effect does not reach significance. Despite significantly decreasing the number of omissions made, ACTH treatment does not bring the number of omissions back to control levels.

**Figure 4:**
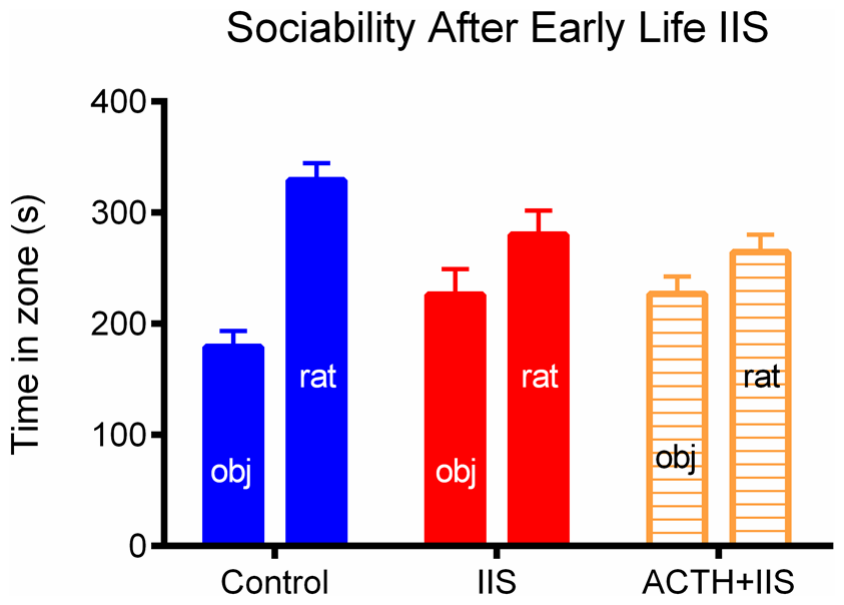
Deficits in sociability are not significantly ameliorated by treatment with ACTH. Sociability as defined by the relative amount of time spent with a novel rat (bars labeled “R”) over a novel object (bars labeled “O”), was also altered by early life IIS. The group by time effect can be seen as a reduction in the ratio of the amount of time spent with the rat compared to the amount of time spent with the object. Control animals (blue) spent a significantly greater proportion of time with the novel rat than the novel object compared to IIS rats (red), and the IIS rats treated with ACTH (orange). There are no significant differences between the IIS and IIS+ACTH groups. Data points on all graphs represent mean ± SEM, N = 9 Control, N = 9 IIS, N = 8 IIS+ACTH.

We next used a test of sociability to determine whether ACTH was able to ameliorate the previously-noted IIS-induced deficits in sociability. Unlike with the attention deficit, treatment with ACTH did not have a significant effect on sociability in our animals (p>0.05, Fig. 4). Both the treated and the untreated IIS groups were not significantly sociable, spending statistically equal amounts of time with the novel rat as the novel object. The control group was significantly sociable, spending more time with the novel rat than the novel object.

Data presented in the behavioral experiments represent pooled data from three separate cohorts of animals. One of these cohorts, containing N = 5 control and N = 5 untreated IIS animals, are in press in a separate publication [12]. The addition of these animals does not alter the outcome of any experiments (see Figs. S1 and S2).

## Discussion

This experimental model of early life IIS was developed to assess the effect of *focal* inhibitory/excitatory imbalance resulting in IIS activity on cognition in order to (1) control for secondary effects of IIS on the PFC through connections from other brain regions (2) control for global brain injury incurred during generalized seizures. While this model may not speak directly to pediatric epilepsies with global IIS activity or frequent generalized seizures, our goal was to understand the long-term cognitive consequence of frequent GABA signaling disruption and resultant epileptiform activity on a single developing brain region whose dysfunction underlies cognitive deficits seen in patients with pediatric epilepsies. We have tailored the time of exposure roughly to the time period of development of the PFC itself. Thus in modeling early life IIS experimentally, we hoped to understand specific cognitive deficits seen in patients with pediatric epilepsies such as Landau Kleffner where seizures are infrequent but epileptiform activity is prevalent. Our subsequent goals, addressed in this study, were to ameliorate some of these cognitive deficits using a pharmacological agent.

To this end, we have examined the role of ACTH treatment on IIS-associated deficits in behavior and found a modest but significant decrease in the number of omissions made during a DNMS task, suggesting ACTH may have a role in improving an attention deficit associated with early life epileptiform activity. Since ACTH is one of the few drugs that has been shown to not only treat the symptoms of epilepsy but also normalize the EEG [13] and is associated with better cognitive outcomes in children with epilepsy [14], it was chosen to address the question of whether pharmacological intervention can improve IIS-associated cognitive deficits. The mechanism of cognitive deficits after early life IIS is often thought to be directly related to the IIS activity itself; however it is also possible that IIS activity initiates deleterious downstream processes that lead to cognitive deficits. In this study, we show that ACTH exerts its effect on the IIS-associated attention deficit through a mechanism that does not involve alteration of the epileptiform activity itself, suggesting IIS-associated cognitive deficits may not be solely related to the aberrant electrical activity. In both our model and a patient population with epilepsy and comorbid cognitive dysfunction, it is likely both the IIS themselves during neurodevelopment and the underlying signaling abnormality that leads to the IIS that cause cognitive problems. To our knowledge, this is the first time *any* improvement in cognitive outcome has been shown in the context of pediatric epilepsy in the absence of any alteration in epileptiform activity.

ACTH has many targets in the brain and peripheral tissues. Melanocortin receptors that bind ACTH directly are located on a variety of different cell types throughout the CNS [15], [16], and have neuroprotective effects [17], [18]. ACTH also leads to downstream upregulation of neurosteroids that act on GABA_A_ receptors, can reduce neuroinflammation [19] and can also downregulate CRH [20], which may be an endogenous proconvulsant in developing brains [21], [22]. The mechanism of action of ACTH in epilepsy is likely a combination of these actions working in concert and it is this wide range of anti-epileptic properties that made it ideal for a first-pass drug for this study.

The selectivity of the ACTH effect on attention over sociability was an unexpected but interesting result**.** This selectivity may be due to a difference in the nature of the sociability versus the DNMS task. The sociability task is a single session task, where there is one outcome measure per animal. This makes the task less sensitive than the DNMS attention measure, which has 5 or more sessions per animal. ACTH does ameliorate the attention deficit, but it does not bring it back to control levels, so it is possible that the change in sociability may be subtle as well. The decreased sensitivity of the sociability task suggests that subtle changes are not measureable with this task.

The suggestion that treating the cognitive deficits may be possible without treating the overt aberrant electrical activity has important implications for the way epilepsies are treated in the pediatric population [23]. Understanding the both the extent to which ACTH can improve cognitive outcomes after early life IIS and mechanism by which ACTH can modulate networks underlying cognition may allow for better treatments for some of the devastating cognitive and psychiatric comorbidities in the future.

## Conclusions

ACTH administration to animals experiencing IIS does not change the features of the IIS. However, ACTH treatment appears to produce some alteration in attention deficits in these animals. This result is deserving of future study in order to determine the extent to which ACTH can alter deleterious cognitive outcomes, but is both surprising and exciting as it suggests that we may be able to treat a subset of cognitive deficits associated with abnormal EEG activity, even when we are unsuccessful at eliminating the EEG abnormalities. Important future directions should focus on understanding full extent to which ACTH can alter cognitive outcomes in this and other animal models, and subsequently understanding the mechanism by which ACTH exerts its effects in order to identify other potentially useful treatments for cognitive deficits in the pediatric epilepsy.

## Supporting Information

Figure S1 **Improvements in attention after ACTH administration.** Working memory as measured by cumulative number of sessions to task completion (**A**) and accuracy in the training session block (**B**) in the DNMS task were not different after IIS, or with treatment with ACTH. However, IIS animals (red) made significantly more omissions per session in the task than their control (blue) counterparts, and this was significantly improved with ACTH treatment (orange) (p<0.05) (**C**). Inset in (**C**) shows the presence of an outlier (circled) in the ACTH+IIS (orange) group. The number of omissions errors per day did not vary based on the difficulty in the working memory component of the task (**D**). Data points on all graphs represent mean ± SEM, N = 4 Control, N = 4 IIS, N = 8 IIS+ACTH.
(TIF)

Figure S2 **Deficits in sociability are not significantly ameliorated by treatment with ACTH.** Sociability as defined by the relative amount of time spent with a novel rat (bars labeled “R”) over a novel object (bars labeled “O”), was also altered by early life IIS. The group by time effect can be seen as a reduction in the ratio of the amount of time spent with the rat compared to the amount of time spent with the object. Control animals (blue) spent a significantly greater proportion of time with the novel rat than the novel object compared to IIS rats (red), and the IIS rats treated with ACTH (orange). There are no significant differences between the IIS and IIS+ACTH groups. Data points on all graphs represent mean ± SEM, N = 4 Control, N = 4 IIS, N = 8 IIS+ACTH.
(TIF)
