# Supplementary figures and images for: Attention Deficit Associated with Early Life Interictal Spikes in a Rat Model Is Improved with ACTH
Source: PLoS One. 2014 Feb 24;9(2):e89812. doi: 10.1371/journal.pone.0089812 (PMC3933669; doi:10.1371/journal.pone.0089812)

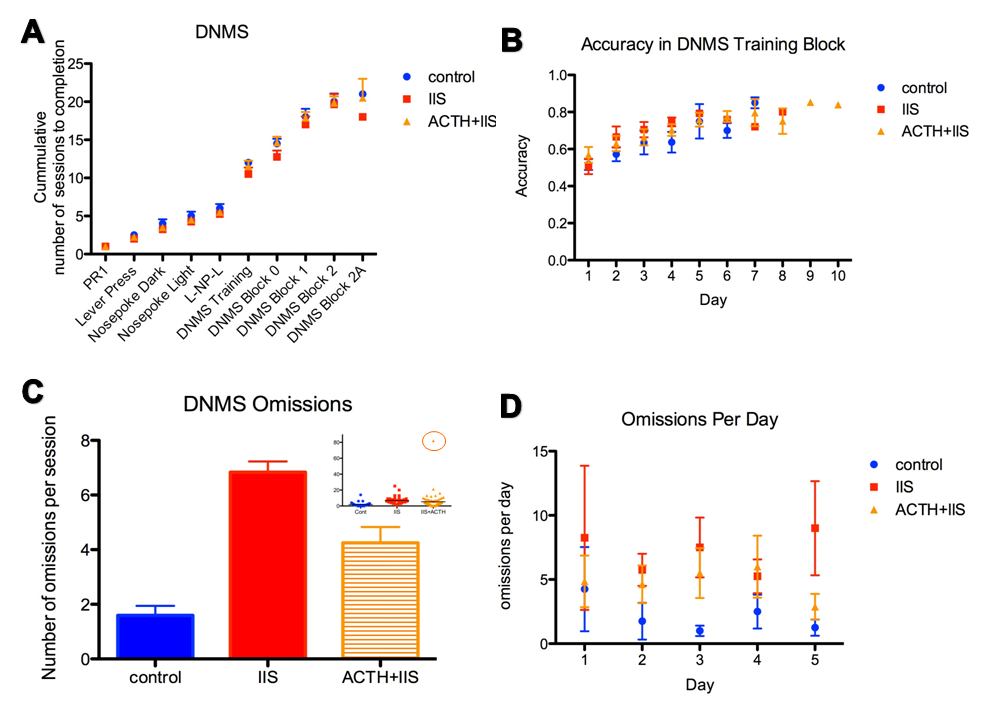

Supplement: Figure S1 — Improvements in attention after ACTH administration. Working memory as measured by cumulative number of sessions to task completion (A) and accuracy in the training session block (B) in the DNMS task were not different after IIS, or with treatment with ACTH. However, IIS animals (red) made significantly more omissions per session in the task than their control (blue) counterparts, and this was significantly improved with ACTH treatment (orange) (p<0.05) (C). Inset in (C) shows the presence of an outlier (circled) in the ACTH+IIS (orange) group. The number of omissions errors per day did not vary based on the difficulty in the working memory component of the task (D). Data points on all graphs represent mean ± SEM, N = 4 Control, N = 4 IIS, N = 8 IIS+ACTH. (TIF) [file pone.0089812.s001.tif]

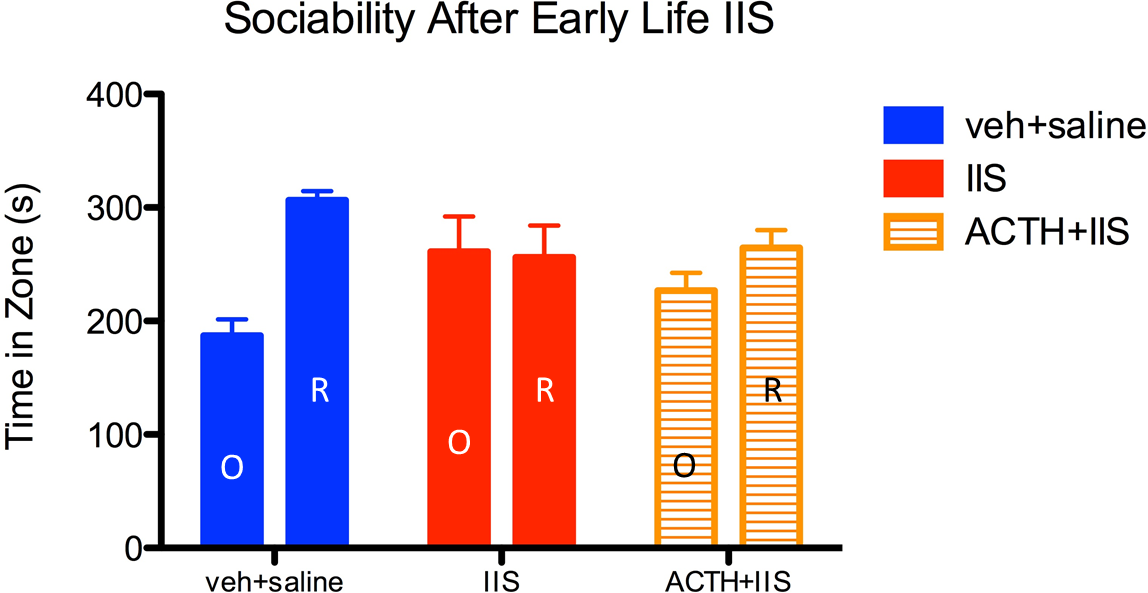

Supplement: Figure S2 — Deficits in sociability are not significantly ameliorated by treatment with ACTH. Sociability as defined by the relative amount of time spent with a novel rat (bars labeled “R”) over a novel object (bars labeled “O”), was also altered by early life IIS. The group by time effect can be seen as a reduction in the ratio of the amount of time spent with the rat compared to the amount of time spent with the object. Control animals (blue) spent a significantly greater proportion of time with the novel rat than the novel object compared to IIS rats (red), and the IIS rats treated with ACTH (orange). There are no significant differences between the IIS and IIS+ACTH groups. Data points on all graphs represent mean ± SEM, N = 4 Control, N = 4 IIS, N = 8 IIS+ACTH. (TIF) [file pone.0089812.s002.tif]
